# Characterization of the complete chloroplast genome of *Veronica arvensis* and its phylogenomic inference in plantaginaceae

**DOI:** 10.1080/23802359.2022.2139162

**Published:** 2022-11-04

**Authors:** Huabing Liu, Wenmiao He, Xiaobing Zhang, Zhimin Jiang, Qi Li, Chen Xia, Hui Wang

**Affiliations:** China Tobacco Zhejiang Industry Co, Ltd, Hangzhou, China

**Keywords:** *Veronica arvensis*, plastid genome, Plantaginaceae, phylogeny inference

## Abstract

*Veronica arvensis*, which is an annual flowering plant in the plantain family Plantaginaceae, has commonly used as a Chinese herbal medicine to treat malaria in China. Here, the complete plastome of *V. arvensis* was successfully assembled based on genome skimming sequencing. The plastome of *V. arvensis* was 149,386 bp in length, comprising a pair of inverted repeats (IR; 24,946 bp) separated by a large single-copy (LSC) region (82,004 bp) and a small single-copy (SSC) region (17,490 bp). The plastid genome encoded 113 unique genes, consisting of 79 protein-coding genes, 30 tRNA genes, and four rRNA genes, with 19 duplicated genes in the IR regions. Six plastid hotspot regions (*trn*H-*psb*A, *trn*K-*rps*16, *atp*I-*rps*2, *ndh*F-*rpl*32, *ccs*A-*ndh*D and *rps*15-*ycf*1) were identified within *Veronica*. Phylogenetic analysis showed that the representative species from *Veronica* was monophyletic. *V. persica* and *V. polita* formed a maximum clade, followed by sister to *V. arvensis*.

For the past three decades, molecular data have been increasingly used to characterize plant diversity, which has had a profound impact on our understanding of plant diversification patterns and phylogenetic relationships (Straub et al. 2012). Plastomes, usually mapped as circular genomes, are increasingly used for reconstructing the angiosperm phylogenetic backbone due to its relatively small size, conserved gene content and simple structure (Liu et al. 2017). Recent advances in sequencing technology have made it easier to obtain the complete plastomes for any plant species, regardless of model or not model organisms (Malé et al. 2015). *Veronica* L. is the largest genus in the flowering plant family Plantaginaceae, with about 500 species distributed in the world (Mazur et al. 2021). However, there were only six plastomes sequenced and submitted to GenBank (retrieved July 2022 from https://www.ncbi.nlm.nih.gov), which hinder to resolve the phylogeny of *Veronica. V. arvensis* Linaeus 1753 (Figure 1), which is native to European, is an annual flowering plant of *Veronica* and usually grown in gardens, pastures, and cultivated land (Baskin and Baskin 1983). According to traditional Chinese medicine, whole plant of *V. arvensis* had been used to treat malaria in Jiangsu and Zhejiang provinces (Zhao 1963). In this study, the plastid genome of *V. arvensis* was firstly sequenced and assembled using genome skimming data, and comparative analyses of plastomes were performed for several representative species to screen the highly divergent regions (plastid hotspot) in the plastome of *Veronica* species.

**Figure 1.**
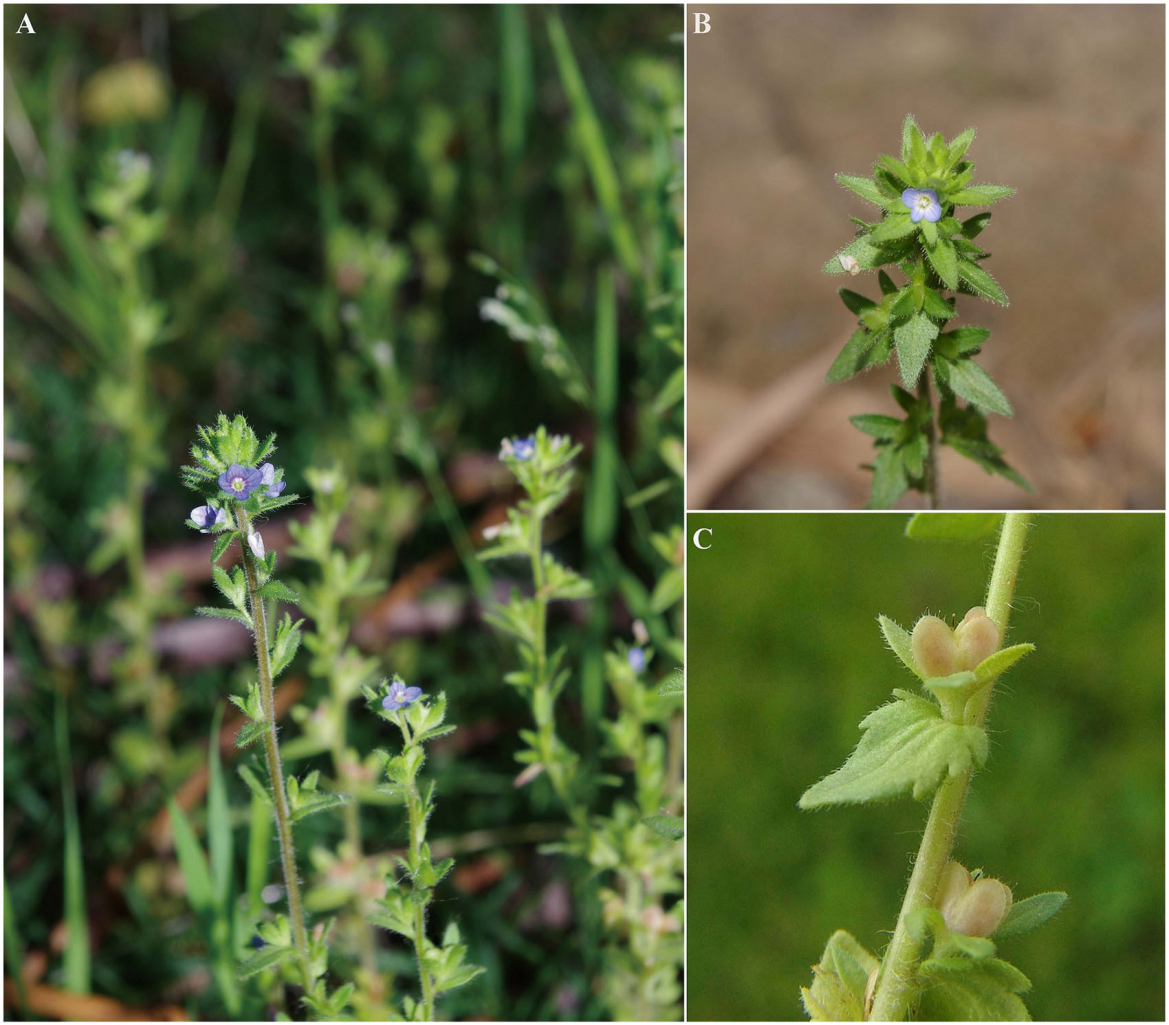
The morphological characteristics of *V. arvensis*. A, B, C showing the photo of whole plant, flowers, and fruits, respectively (photos taken by Qi Li at Henan University).

Fresh and healthy leaves of *V. arvensis* were collected in Jinming campus of Henan University, Kaifeng (China; 114°18′14.23″E, 34°49′08.72″N). Field work for collecting plant sample did not require a specific permission, and no protected or endangered species were involved. A voucher specimen (*LQ20220401; Qi Li,* lqzjtobacco@163.com) was deposited in the Herbarium of Henan University (HHN). Total genomic DNA was extracted using Plant DNAzol Reagent (LifeFeng, Shanghai) according to the manufacturer’s protocol with ∼6 mg of the fresh leaf tissue. Illumina paired-end libraries (150 bp read length) were prepared from sheared genomic DNA (fragment size ≤800 bp) and sequenced on an Illumina HiSeq X10 by Beijing Genomics Institute (BGI, Wuhan, China). The raw reads with the adapters trimmed were filtered by quality with Phred scores of 30 or less implemented in the CLC-quality trim tool, and the filtered reads were assembled using GetOrganelle pipeline (Jin et al. 2020). Combined commands were employed in the pipeline to recruit plastid-like reads using Bowite2 (Langmead and Salzberg 2012) and assembled them using SPAdes (Bankevich et al. 2012). The software Geneious R11 (Biomatters, Auckland, New Zealand) was used to annotate the plastome with *V. eriogyne* H. Winkl. 1922 (GenBank accession number: MZ323109) as a reference. The plastome map of *V. arvensis* was drawn using the OGDRAW program (Lohse et al. 2013). The genome sequence was registered into GenBank with the accession number ON461915. Multiple alignments of the seven plastomes of *Veronica* species (the species name and GenBank accession numbers were seen in Figure 1) were carried out using MAFFT v7.017 (Katoh and Standley 2013) to screen for plastid hotspot regions in *Veronica*, and nucleotide diversity (*Pi*) was determined using DnaSP v.5.0 (Librado and Rozas 2009). Maximum likelihood (ML) was performed to estimate the phylogeny for 27 plastome sequences of Plantaginaceae species, with two *Osmanthus* Lour. species as outgroups. ML analysis was implemented at the CIPRES Science Gateway v3.3 (Miller et al. 2010) using RAxML v8.1.11 with the optimal substitution model (GTR + I+G) determined by the software jModel Test v2.1.4 (Posada 2008), 1000 bootstrap iterations were conducted with other parameters using the default settings.

The total of 30,763,654 paired-end reads were generated from genome skimming sequencing for *V. arvensis*, and 7,053,345 reads were trimmed due to low quality. The plastome of *V. arvensis* was 149,386 bp in length and shared the common quadripartite structure of most angiosperms (Figure 2). The genome comprised two copies of IR (24,946 bp each) separated by a LSC region (82,004 bp) and a SSC region (17,490 bp). There were 113 unique genes including 79 protein-coding genes, 30 tRNA genes and four rRNA genes annotated in the plastome of *V. arvensis*, with 19 duplicated genes in the IR regions. In addition, we generated 131 loci (70 genic and 61 inter-genic spacer regions) within *Veronica* with more than 200 bp in length and the nucleotide variability (*Pi*) values were ranged from 0.0009 to 0.1367 (Figure 3). Six of these variable loci (*Pi* > 0.09) including *trn*H-*psb*A, *trn*K-*rps*16, *atp*I-*rps*2, *ndh*F-*rpl*32, *ccs*A-*ndh*D and *rps*15-*ycf*1 showed high level of intrageneric variation. The phylogeny revealed that all the *Veronica* species formed a maximum supported (bootstrap value = 100) clade, and *V. arvensis* was sister to the clade formed by *V. persica* Poir. 1808 and *V. polita* Fries 1819 within the genus *Veronica* (Figure 4).

**Figure 2.**
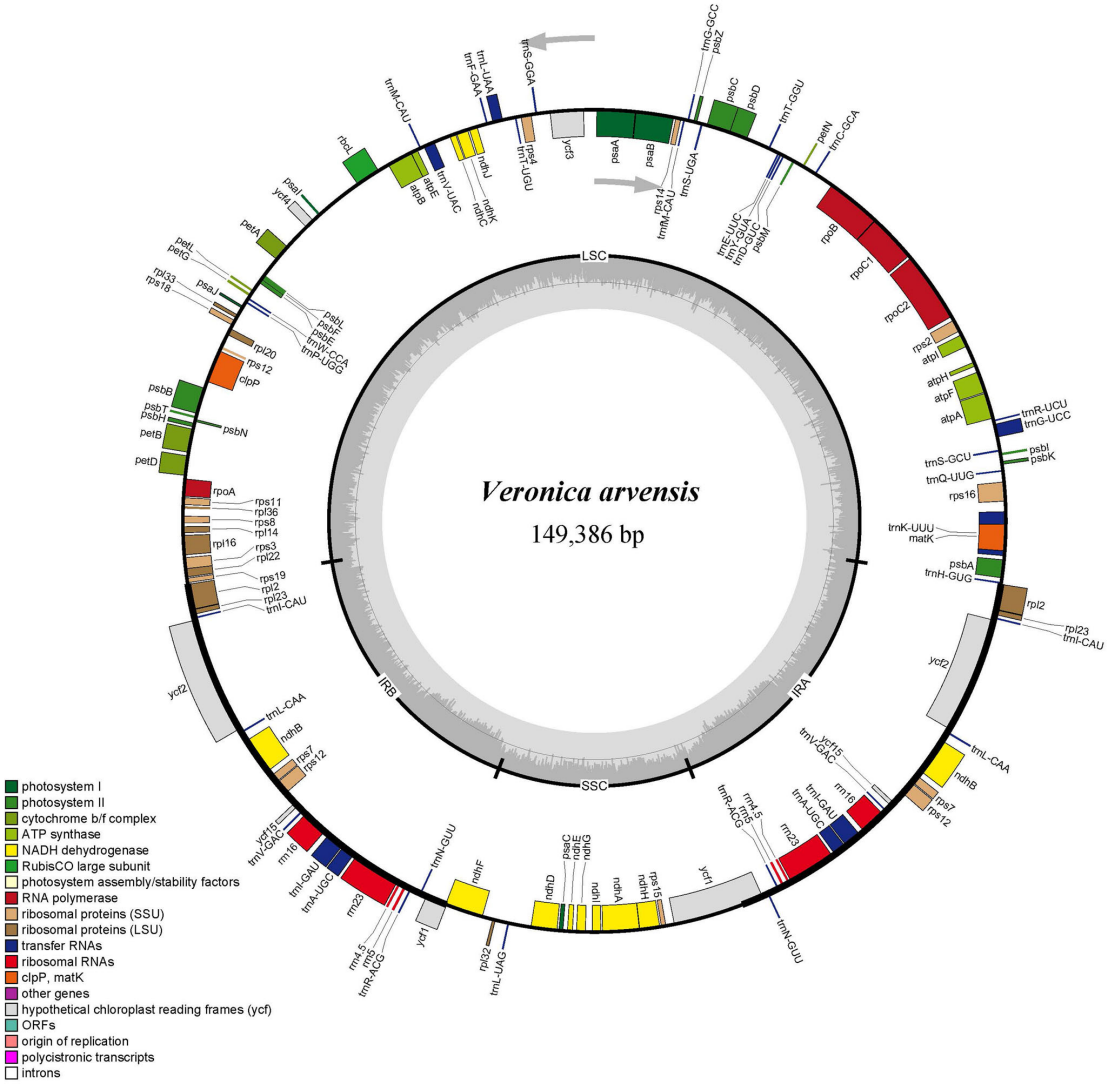
Plastid genome map of *V. arvensis*. The genes inside and outside of the circle are transcribed in the clockwise and counterclockwise directions, respectively.

**Figure 3.**
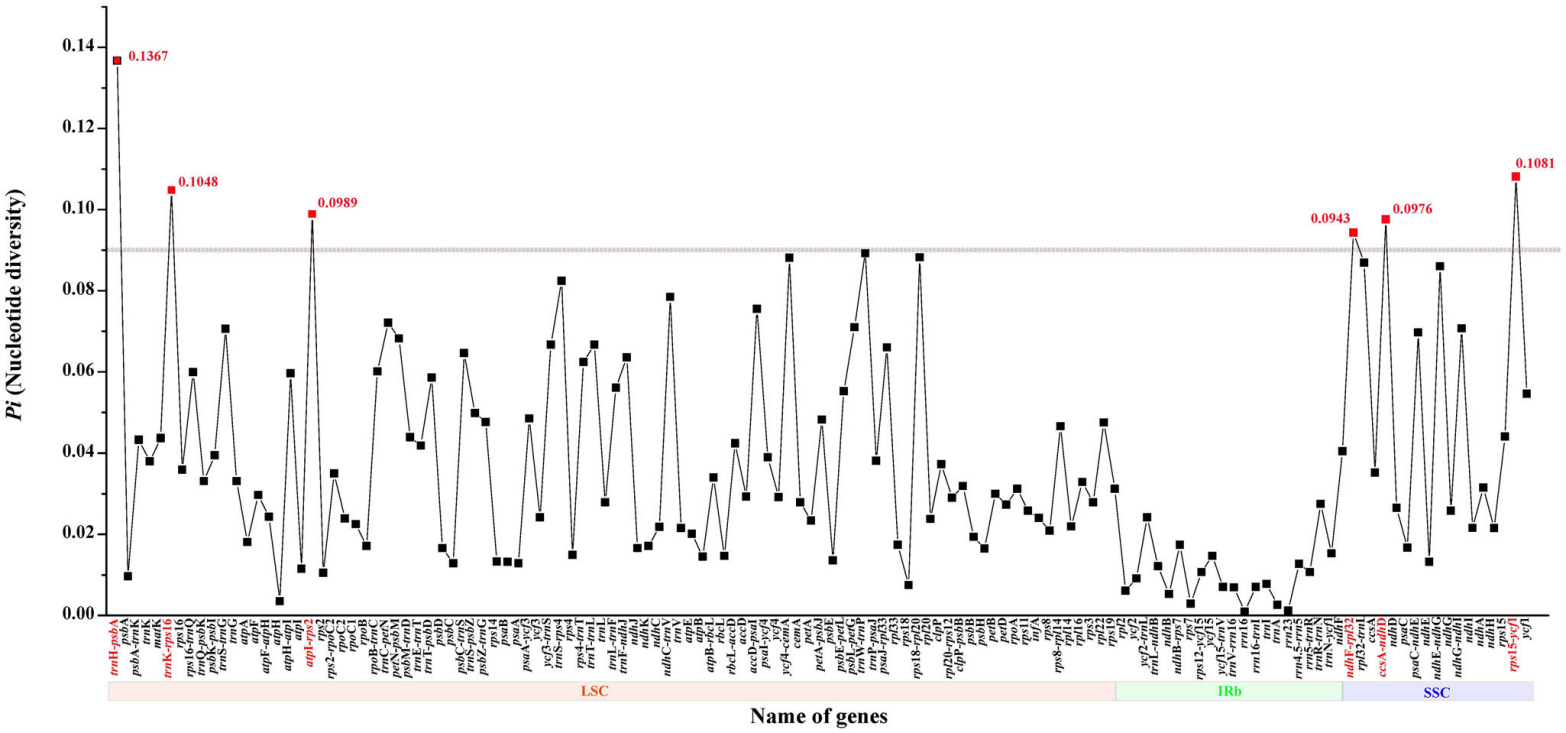
Comparative analysis of the nucleotide variability (*Pi*) values among seven *Veronica* species.

**Figure 4.**
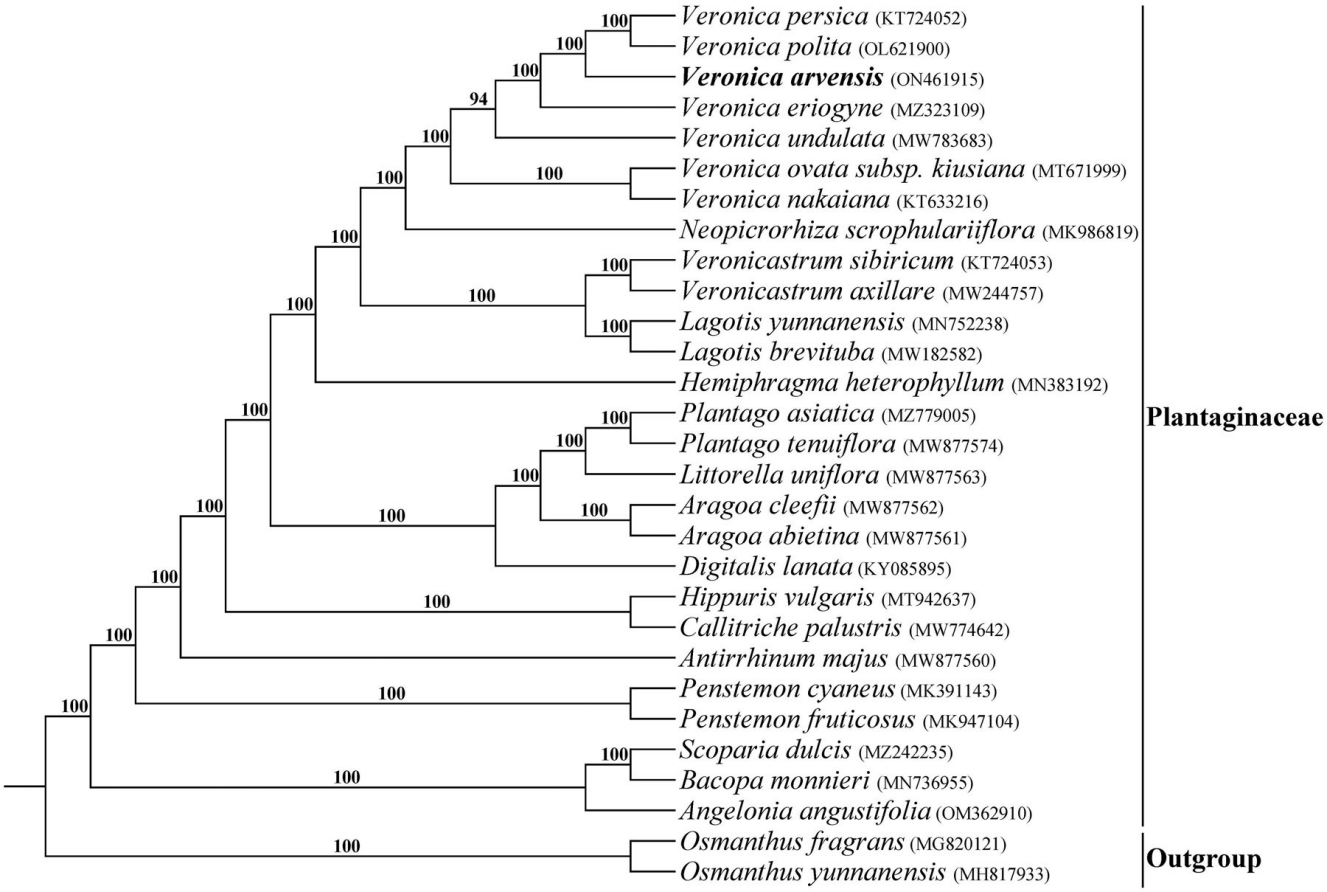
Phylogenetic tree of Plantaginaceae inferred using maximum likelihood (ML) based on complete plastome sequences. Numbers above the branches represent bootstrap values from ML analyses.

In the last ten years, high-throughput sequencing technologies have made it easier to obtain genomic data for any plant species, regardless of model organism status (Dodsworth 2015). The term ‘genome skimming’ introduced by Straub et al. (2012), is a method of reducing sampling of low-coverage genome data by selective assembly of high-copy portions (plastome, mitogenome and repetitive elements). In many previous studies, complete plastomes have been successfully assembled using genome skimming data and subsequently used for phylogenetic reconstruction (Malé et al. 2015; Liu et al. 2017). Here, we assembled and reported the plastome of *V. arvensis* for the first time based on genome skimming data, the genome shared a common quadripartite structure similar to the majority of other angiosperms. Among the seven representative *Veronica* species, *V. arvensis* exhibited the smallest genome size and *Veronica nakaiana* had the largest one. The genome size variations of the plastomes were purely ascribed to the length differences of intergenic and intronic regions. Comparative plastome analysis showed that *ycf15*, which has been paid great attention to its function in previous studies (Raubeson et al. 2007; Shi et al. 2013), had been pseudogenized in *V. persica* and *V. polita*. The phylogeny inference reconstructed from complete plastomes revealed all the *Veronica* species formed a monophyletic clade and the branches were supported with higher bootstrap values than the results based on plastid, nuclear ribosomal and nuclear low-copy DNA (Albach and Meudt 2010), which proved that complete plastome sequences were more effective for the phylogenetic reconstruction of *Veronica*. Furthermore, six plastid hotspot regions identified among *Veronica* species will be useful in future studies reconstructing the phylogenetics relationships and characterizing the population genetics of this genus.

## Data Availability

The genome sequence data that support the findings of this study are openly available in GenBank of NCBI at https://www.ncbi.nlm.nih.gov/under the accession no. ON461915. The associated Bio-Project, SRA, and Bio-Sample numbers of the raw sequence data for assembling the plastome are PRJNA835916, SRR19134697, and SAMN28125189, respectively.
